# Effectiveness of the Monte Carlo method in stereotactic radiation therapy applied to quasi-homogenous brain tumors

**DOI:** 10.18632/oncotarget.7280

**Published:** 2016-02-09

**Authors:** Ki Mun Kang, Bae Kwon Jeong, Hoon Sik Choi, Jin Ho Song, Byung-Do Park, Young Kyung Lim, Hojin Jeong

**Affiliations:** ^1^ Department of Radiation Oncology, Gyeongsang National University School of Medicine and Gyeongsang National University Hospital, Jinju, Republic of Korea; ^2^ Institute of Health Sciences, Gyeongsang National University, Jinju, Republic of Korea; ^3^ Department of Radiation Oncology, Samsung Changwon Hospital, Sungkyunkwan University School of Medicine, Changwon, Republic of Korea; ^4^ Proton Therapy Center, National Cancer Center, Goyang, Republic of Korea

**Keywords:** radiosurgery, radiotherapy, Monte Carlo, ray tracing, tissue heterogeneity

## Abstract

This study was aimed to evaluate the effectiveness of Monte Carlo (MC) method in stereotactic radiotherapy for brain tumor. The difference in doses predicted by the conventional Ray-tracing (Ray) and the advanced MC algorithms was comprehensively investigated through the simulations for phantom and patient data, actual measurement of dose distribution, and the retrospective analysis of 77 brain tumors patients. These investigations consistently showed that the MC algorithm overestimated the dose than the Ray algorithm and the MC overestimation was generally increased as decreasing the beams size and increasing the number of beams delivered. These results demonstrated that the advanced MC algorithm would be inaccurate than the conventional Raytracing algorithm when applied to a (quasi-) homogeneous brain tumors. Thus, caution may be needed to apply the MC method to brain radiosurgery or radiotherapy.

## INTRODUCTION

The CyberKnife is a dedicated system for radiosurgical treatments [1, 2], including stereotactic radiosurgery [3] and stereotactic body radiotherapy [4–6], and incorporates two different dose calculation algorithms: Raytracing (Ray) algorithm based on the effective path length (EPL) correction scheme and Monte Carlo (MC) algorithms [2, 7, 8]. The accuracy of the algorithms incorporated in CK has been widely investigated through numerous studies [9–15]. These studies have consistently reported that the Ray algorithm has limited applicability to heterogeneous tumors, and the limitations could be improved by using the MC method [9–13]. Thus, the use of the MC algorithm, though significantly less efficient in calculation, strongly recommended for treating the tumors in heterogeneous regions, such as the lungs [13].

However, the effectiveness of the MC methods for homogeneous tumors has been overlooked. Only a few studies have compared the dosimetric differences predicted by the Ray and MC methods at sites other than the lungs [16]. Although non-negligible differences between the Ray and MC calculations have been shown in the studies, no explanations for these differences have been proposed and it is not clear whether one algorithm is more accurate than the other in the homogeneous regions.

In the present study, the nature of the Ray and MC algorithmic differences with respect to predicted dose distributions and the accuracy of each algorithm in (quasi-) homogeneous environment were investigated in application to CK using phantom simulations, a retrospective analysis of actual patient plans, and film-based measurements. The actual patient plans analyzed here were selected from brain tumor cases because the brain tumor is one of the most common indications for radiosurgical treatment, and more importantly, is generally in more homogeneous region compared to tumors sited in other places. The present investigations consistently showed that the MC algorithm provided rather limited accuracy compared to the Ray algorithm in (quasi-) homogeneous regions, and the limitations became significant as the radiation beams more overlapped within the unit targeted area.

## RESULTS

### Single beam properties: virtual water phantom study

**Figure 1:**
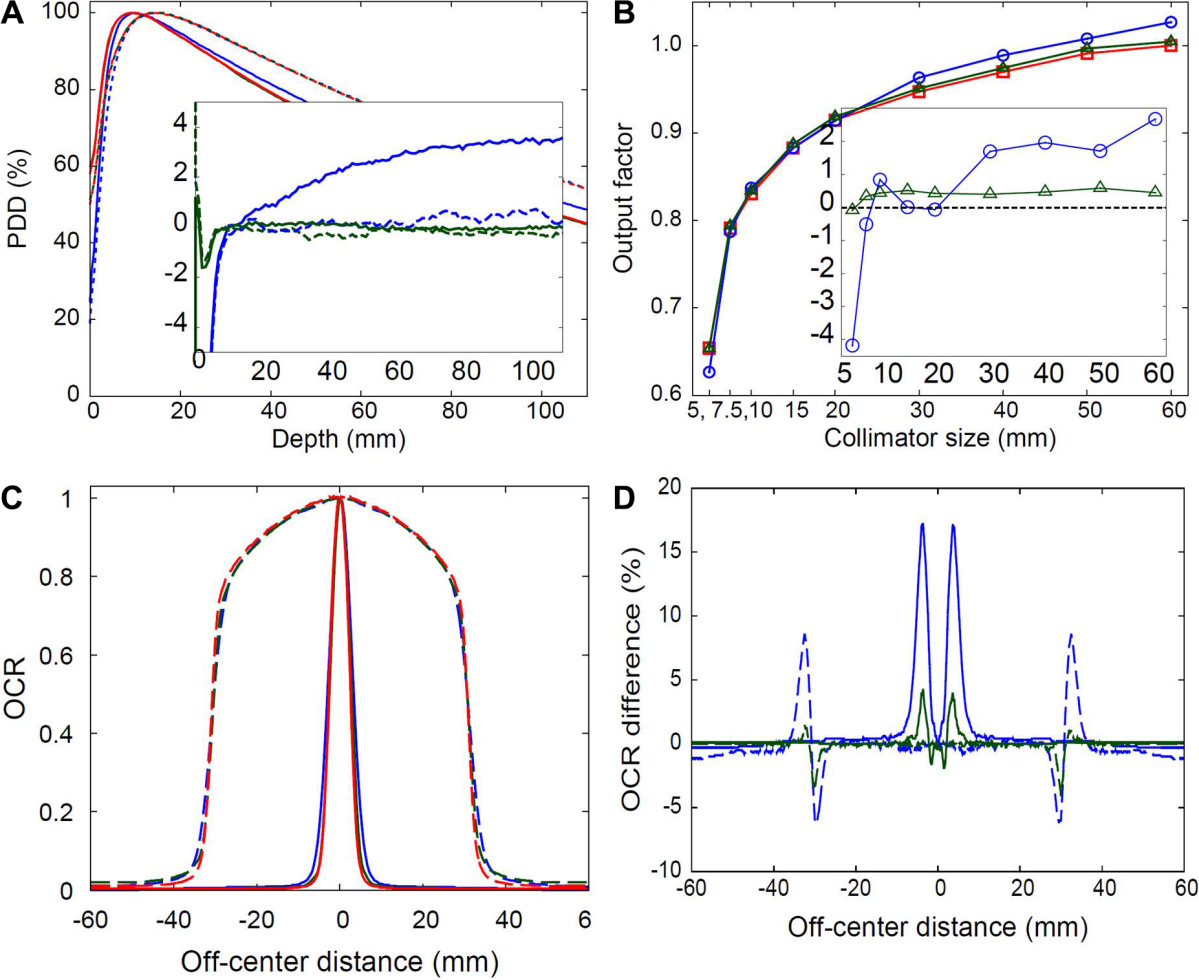
(**A**) The depth-dose, output factor, and (**C** and **D**) OCR dose profiles calculated with MC (blue lines) and Ray (red lines) algorithms, respectively, for the 60-mm (solid line) and 5-mm (dotted line) beams and those for measured results (green lines). The inset figures in (A) and (**B**) are the percent differences of PDD and output factor, respectively, for MC (blue lines) and Ray (green lines) results relative to the corresponding measurements.

The depth–dose curves and the off-center ratios (OCR) profiles were calculated for nine different beam sizes ranging from 5 mm to 60 mm in diameter using the Ray and MC algorithms, but only the two extremes (the smallest 5-mm and the largest 60-mm beams) of the results are presented in Figure 1.

The depth–dose curves obtained from the Ray calculations reproduced well the measured results for all beams examined, whereas the MC results deviated partially from the corresponding measurements, depending on the beam size (Figure 1(A)). The differences were relatively quantified in Figure 1(B) according to the doses at the central 5-cm depth point or the output factor. A comparison to the measurements revealed that the Ray doses agreed with the measured values to within 0.6%, whereas the MC doses were either overestimated or underestimated, depending on the beam size. The MC dose was overestimated by at most 2.7%, relative to the corresponding measurement, with the largest 60-mm beam, and was underestimated by at most −4.2% with the smallest 5-mm beam. At intermediate beam sizes, the difference between the MC calculated results and the measured doses varied gradually between the two extremes (See inset in Figure 1(B)).

Disagreement between the MC doses and the measurement doses was observed in the lateral dose profiles, as shown in Figure 1(B). The lateral profiles obtained from the MC calculations were consistently broader compared to the corresponding measurements as well as the Ray calculations. The maximal difference between the MC-simulated profile and measured profile obtained from each irradiated beam was 6%–9%. In sharp contrast, the Ray-simulated profiles agreed well with the measurements within 2% at all calculation points for all irradiated beams, as displayed in Figure 1(D).

**Figure 2:**
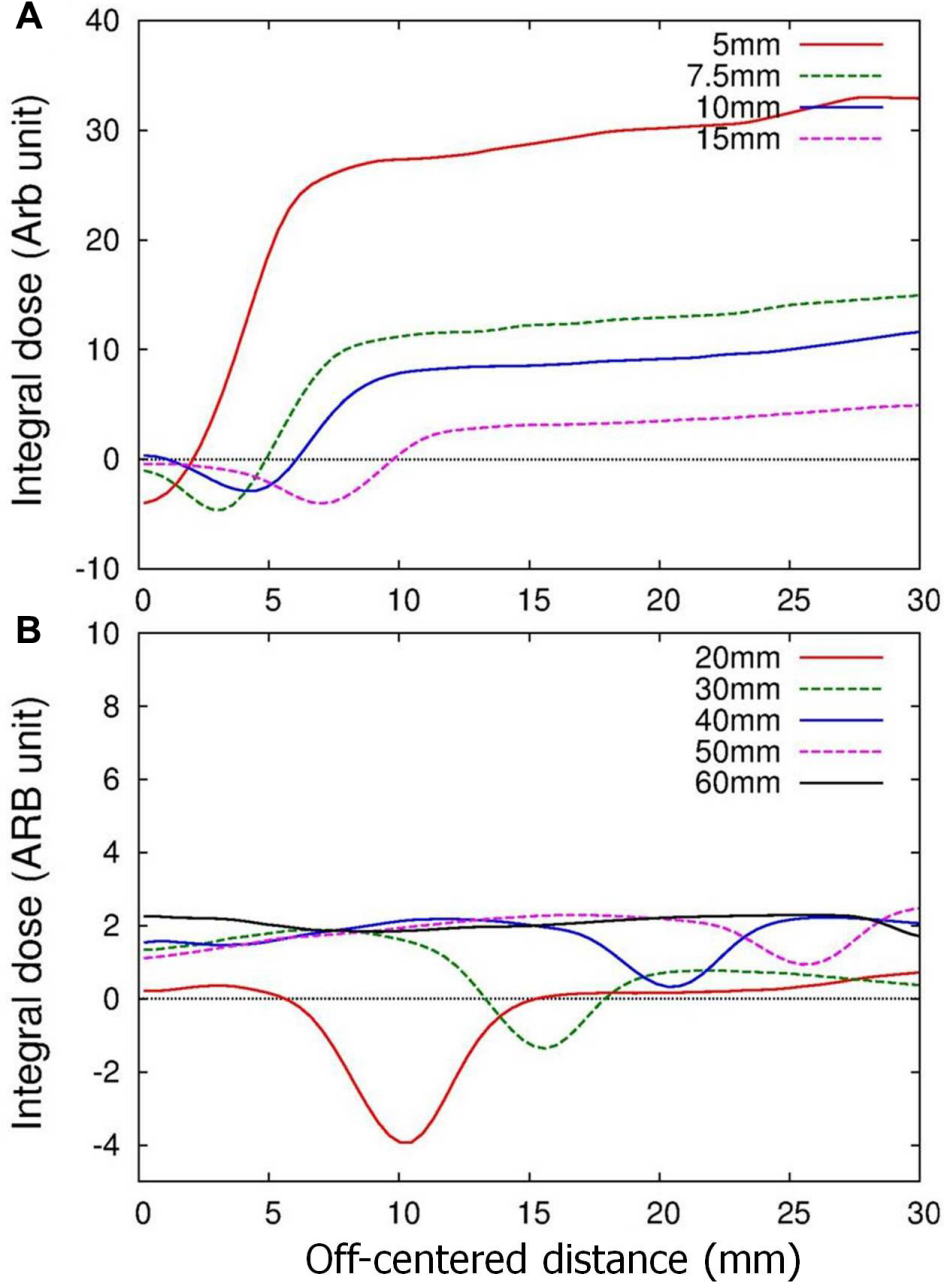
Integral differences of MC relative to Ray doses as a function of off-centered distances for the beams used in the virtual-water phantom study

The difference between the MC and Ray doses obtained from each irradiated beam was integrated over the referenced 5-cm depth plane under the assumption of radial beam symmetry, as

$$\int_{0}^{r^{\prime }}\delta (r)\cdot 2\pi r\cdot dr,$$

where *δ(r)* represents the MC dose difference relative to the Ray dose at an off-centered distance of “*r*”. The results were plotted as a function of the off-centered distance (*r*) in Figure 2 and revealed that the integral difference always converged to a positive value, demonstrating a higher integral dose predicted by the MC than by the Ray calculations. The integral difference was greatest with the smallest 5-mm beam and gradually decreased as the nominal beam size was increased (Figure 2(B)).

### Dose differences in the head phantom

**Figure 3:**
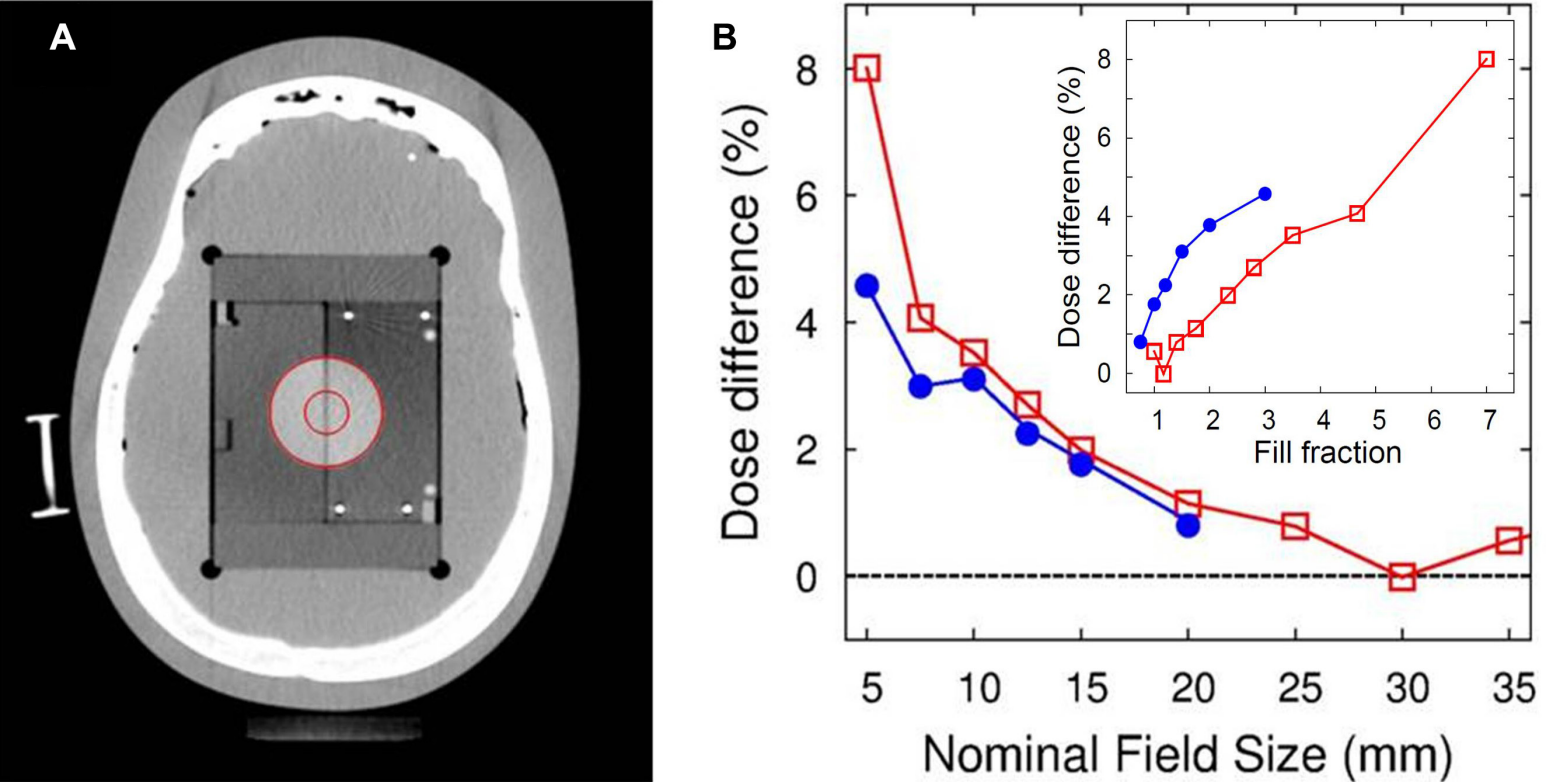
(**A**) Axial slice CT image for the anthropomorphic head phantom, where two spherical virtual gross tumor volumes (vGTV) were drawn (red circles), and (**B**) the differences of MC relative to Ray doses as a function of nominal beam size for the larger (open squares) and the smaller (filled circles) vGTVs, respectively. The inset in (B) is the dose difference as a function of fill fraction or the ratio between tumor diameter and nominal beam size.

Figure 3(A) shows an axial slice of the computed tomography (CT) scan of a head phantom used in the planning study, where two virtual gross tumor volumes (vGTVs) were delineated. The differences in the mean doses predicted by the MC and Ray calculations for each plan and for each vGTV are plotted in Figure 1(B) as a function of the nominal beam size or the size of the fixed collimator. The results showed that the MC calculation commonly overestimated the dose relative to the Ray calculation, and the overestimation grew much significantly when using the smaller beams. The differences between the mean vGTV doses were maximal for the smallest 5-mm beam, with the differences of 8.01% for the larger vGTV (35 mm sphere) and 4.58% for the smaller vGTV (15 mm sphere), respectively. The differences generally decreased with increasing beam size and reached values of 0.4% and 0.5% when using the largest 35-mm and 20-mm beams for each of the larger and smaller vGTVs, respectively.

### Gamma evaluation

**Figure 4:**
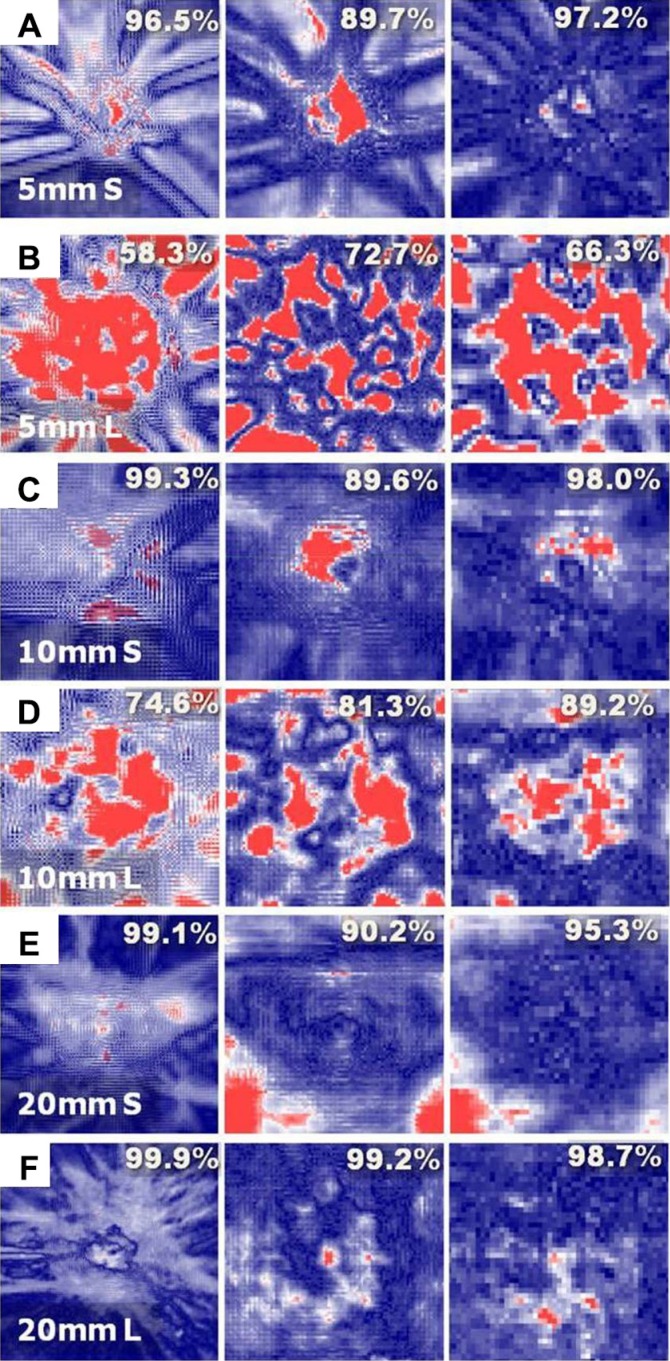
Gamma comparisons between the MC- and Ray-simulated dose distributions (left), between the MC-simulated and measured dose distributions (middle), and between the Ray-simulated and measured dose distributions (right), respectively, for (**A**) *5 S*, (**B**) *5 L*, (**C**) *10 S*, (**D**) *10 L*, (**E**) *20 S*, and (**F**) *20 L* plans for the head phantom shown in Figure 3A. In each Figure, the gamma passing rate with the 2 mm/3% acceptance criterion was given in percentage.

Figure 4 shows comparisons of the gamma results obtained between the Ray and MC calculations (left column), between the measurements and the MC calculations (middle column), and between the measurements and the Ray calculations (right column), respectively, for each treatment plan for smaller and larger vGTVs and with nominal beam sizes of 5 mm, 10 mm, and 20 mm, respectively, *i.e.*, the *5 S, 5 L, 10 S, 10 L, 20 S*, and *20 L* plans.

When evaluating with the 2 mm/3% acceptance criterion, the gamma values between the Ray and MC simulations applied to the *5 L* and *10 L* plans were largely disagreed (gamma passing rates of 58.3% and 74.6% for the *5 L* and *10 L* plans, respectively), whereas other simulation results generally agreed well with one another and yielded gamma passing rates exceeding 95% (96.5% for *5 S* and > 99% for *10 S, 20 S, 20 L*).

The accuracies of the algorithms in reproducing the actual dose were evaluated by comparing the calculated Ray and MC dose distributions to the corresponding measured distributions. The results revealed that the Ray calculations provided better agreement with the measurements compared to the MC calculations. The gamma passing rates calculated between the Ray-simulated and measured dose distributions exceeded the corresponding values obtained between the MC-simulated and measured dose distributions by 5–8% for the *5 S, 10 S, 10 L*, and *20 S* plans, and were almost equal within 0.5% for the *20 L* plan. Only the *5 L* plan yielded a 6% lower passing rate for the Ray algorithm compared to the MC algorithm.

### Dose differences in the actual patient plans

**Figure 5:**
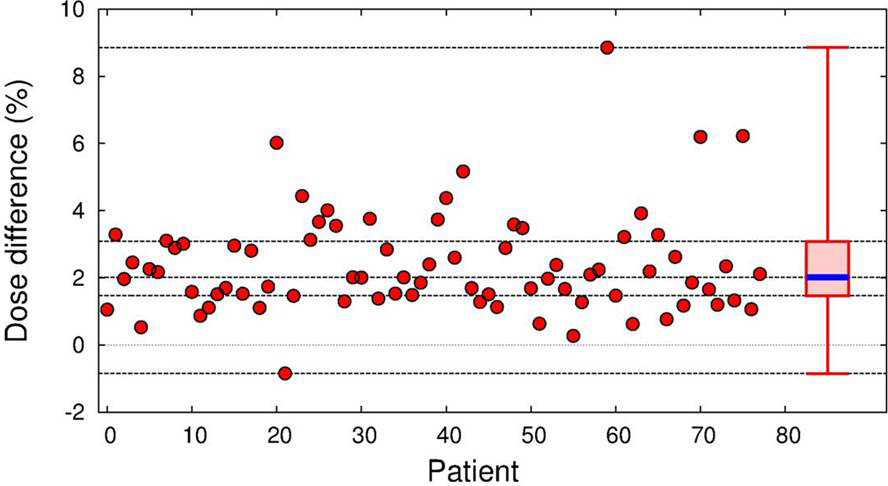
Distribution of dose differences of MC relative to the Ray calculations in the actual 77 patient plans The median, first and third quartiles, maximal, and minimal differences were given by the thick dashed lines.

Figure 5 shows the distributions of the mean MC dose relative to the mean Ray dose (Δ) for the 77 actual patient plans. The differences were always positive with only one exception, indicating that the MC algorithm predicted a larger dose than the Ray calculation. The overall differences ranged from −0.8% to 8.9%, and the median difference was 2.0%. The smallest difference (−0.84%) was found in a trigeminal neuralgia case, which featured the smallest tumor size (*D_avg_* of 5.9 mm) and was treated with the smallest effective beam size (*FS_eff_* of 6.8 mm) among those in the actual treatment plans examined here. The fill fraction (ν), i.e., the ratio between *D_avg_* and *FS_eff_*, for the patient was 0.87. On the other hand, the greatest difference was observed in a metastatic brain tumor case, in which *D_avg_*, *FS_eff_*, and ν were 30.4 mm, 9.4 mm, and 3.3, respectively. The ν for this plan was the largest among the actual patient plans due to the complex tumor shape in the case.

**Figure 6:**
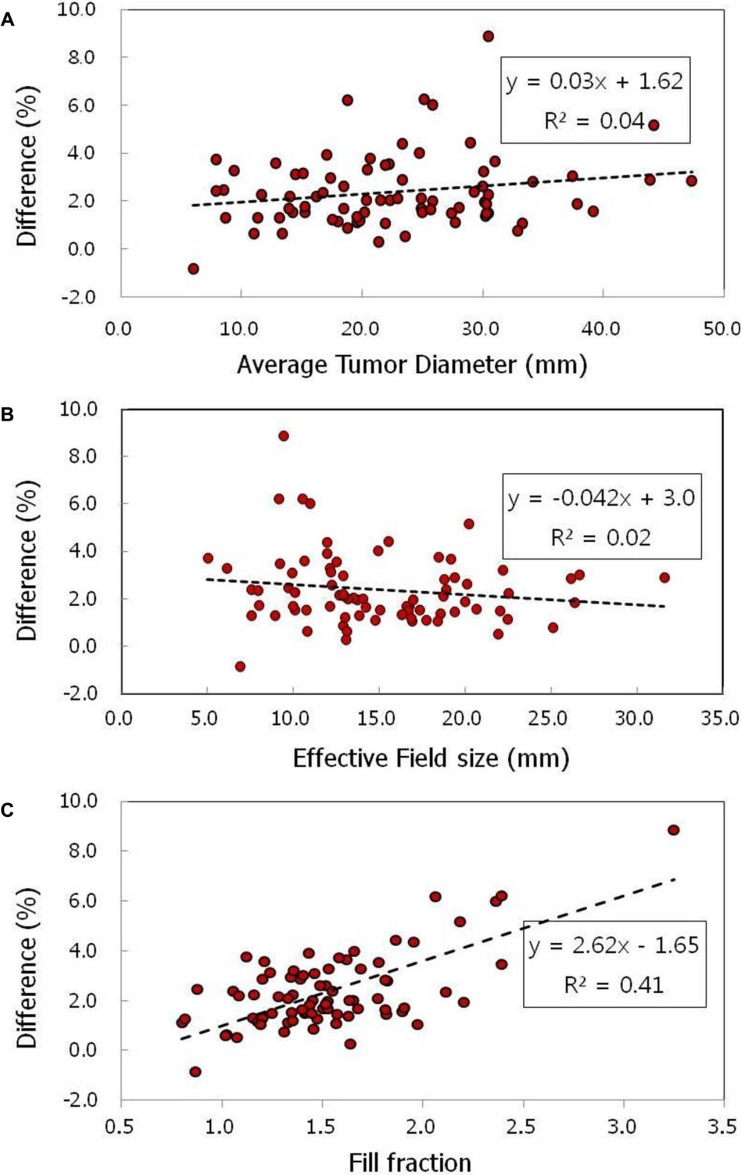
Dose differences of MC relative to the Ray calculations for the 77 actual patients plans as a function of (**A**) average tumor diameter (*D_avg_*), (**B**) effective field size (*FS_eff_*), and (**C**) fill fraction (*ν*), respectively. In each Figure, the regression line, equation, and coefficient of determination (R^2^) obtained from the linear regression analysis were given.

The differences between the mean planning tumor volume (PTV) doses (Δ) in the individual plans are plotted in Figure 6 as a function of *D_avg_*, *FS_eff_*, and ν, respectively, to examine the relationship between the factors using a linear regression method. The result revealed that the dose difference, Δ, was not meaningfully related to *D_avg_* and *FS_eff_*, with the coefficient of determinations (R^2^) less than 0.1. The dose difference was, however, moderately related to ν, with an R^2^ of 0.41.

## DISCUSSION

The MC dose was consistently overestimated compared to the Ray dose in the planning studies examined here, which focused on homogeneous and quasi-homogeneous environments. The simulations of the single-beam characteristics of the CyberKnife beam using a virtual water phantom revealed that this overestimation arose from broadening in the lateral dose profile or the penumbra width in the MC calculation (See Figure 1(C) and 1(D)). Although the central axis depth dose in the MC calculations was underestimated in a subset of the irradiated beams, i.e., beams smaller than 30 mm in diameter (Figure 1(A) and 1(B)), the lateral overestimation by the MC algorithm outweighed the underestimated depth dose. These results were confirmed by integrating the differences between doses predicted by the MC and Ray calculations, as displayed in Figure 2, where the integrated differences consistently converged to positive values and yielded larger integral doses in the MC compared to the Ray calculations. Furthermore, the integral dose difference tended to increase with decreasing beam size, although the depth dose was underestimated by the MC as the beam size decreased.

The phantom planning study results, obtained using a quasi-homogeneous head phantom (Figure 3(A)), were consistent with the simulation results obtained from the virtual water phantom. As shown in Figure 3(B), the mean vGTV dose predicted by the MC calculations always exceeded the value predicted by the Ray calculations, and the difference tended to be increased as the nominal beam size decreased and as the tumor size increased.

The gamma index analysis supported the beam size dependence of the differences in dose predicted by the MC and Ray calculations. The agreement between the gamma values obtained from simulated Ray and MC dose distributions improved as the beam size increased or as the tumor size decreased. In other words, the two algorithms tended to agree for small ratios between the tumor size and the beam size (Figure 3). A comparison between the gamma values obtained from the simulated and measured dose distributions revealed that the Ray calculations generally agreed better with the measurements than with the MC calculations, suggesting better accuracy for the Ray than for the MC algorithms in predicting the actual dose delivered to a (quasi-) homogenous environment.

The present results of large dose and lower accuracy in the MC dose compared to the Ray dose contrasted sharply with previous results obtained in heterogeneous lung cancers [9–13]. Chan *et al.* found that the Ray algorithm greatly overestimated the dose, compared to the MC algorithm, by 13 ± 22% [12]. Similar results were reported in other literatures for SBRT of lung cancer [9–13]. Furthermore, unlike the present results, it has been found better accuracy in the MC algorithm than in the Ray algorithm for heterogeneous media, as validated in previous experimental studies [13, 14].

The contrasting effectiveness of the MC and Ray algorithms in homogenous and heterogeneous media could be explained in terms of the inherent differences between the Ray and MC algorithms with respect to dose estimation. The Ray algorithm interpolates the raw beam data measured in water using a simple correction for the beam geometry and for the beam attenuation along the central beam axis [11]. This algorithm, therefore, is largely limited to be applied to heterogeneous media, such as the lung, because the beam transport characteristics are strongly altered in heterogeneous media compared to the corresponding characteristics in reference water. The simple Ray algorithm could be applied successfully, however, to (quasi-) homogeneous media in which the basic beam transport characteristics are not significantly changed from those obtained in a reference water environment [7, 16].

Unlike the Ray, the MC algorithm numerically estimates the dose distribution for an incident photon beam using the raw beam data only to parameterize the beam geometry and energy spectrum [7, 8]. An appropriately parameterized MC algorithm could provide more reliable results in a heterogeneous environment, such as the lung, compared to the simple Ray algorithm. Caution must be taken, however, because calculation errors can appear in commercial MC algorithms due to use of approximation techniques in estimating the local dose deposition [7, 8, 17–19]. The MC algorithm incorporated in CyberKnife also uses the approximate techniques [7, 8], where multiple photon scatterings are simplified so that only one scattered radiation is generated for every incident photon and is transported only along the averaged trajectory. This approximation may induce an error for scattered radiation dose and the error may increase in the region of increasing scattered radiations, such as penumbra region. It might be, although not entirely clear, one of the possible reasons for discrepancy between the MC-simulated and measured dose distributions (See Figure 1). Considering the fact that the OCR profile is typically broadened in an actual measurement due to the finite size of the detector (active area of 1 mm^2^ for PTW 60012 diode detector used here) [20], the broadened profiles obtained from the MC algorithm relative to the measurement results may arise from overestimated calculation errors, rather than from underestimated measurement errors. Similar pattern of calculation error for MC algorithm also can be found in the previous literature [18, 19], where the calculation error was much bigger at the penumbra region compared to the central axis region.

In a conventional three-dimensional treatment regimen, this type of broadening error may not significantly affect to the tumor dosage because the dosimetric error for each beam is not significant and the erroneous region is generally located outside the target volume. The situation, however, is significantly altered in CyberKnife-based radiotherapy, in which multiply segmented beams are strongly overlapped within a target volume, resulting in the direct influence of lateral broadening effect on tumor dosage. This type of calculation error may become more pronounced as a greater number of beams overlap within unit target area or as the density of subfield overlap increases. Under such circumstances, the ratio between the target volume and the average field size of the delivered beams increases. This explanation agreed well with the present results obtained from the phantom plans (inset in Figure 3) and the actual patients’ plans (Figure 6), in which the MC dose overestimate was found to considerably depend on the fill fraction (ν).

## MATERIALS AND METHODS

### Beam data measurement

The CyberKnife system version 8.5.0 (Accuray, Inc., Sunnyvale, CA) was used in the study. The beam data for all collimated beams from the CyberKnife system were acquired using the small size E-type diode detector (PTW 60012, PTW, Freiburg, Germany) [21]. The diode detector used in the study was reported to have sufficient accuracy for beam data measurement (measurement precisions of ∼1.25% for absolute dosimetry and ∼0.15% for relative dosimetry, respectively) [22].

The central axis tissue-phantom ratios (TPR) were measured at varying depths ranging from 0–300 mm while keeping the source-to-detector distance at 800 mm. The measured TPR data were normalized to unit at depth of maximal dose (15 mm). The OCR of CK beams were collected at five typical depths (15, 50, 100, 200, and 300 mm). The relative output for each beams collimated by the fixed and IRIS collimators were estimated at central 50-mm depth in water phantom relative to the 60-mm fixed collimator output. The measured beam data set were cross-checked by the vendor and confirmed that our beam data were well agreed with the vendor's golden data as well as those of other institutions.

### Virtual water phantom study: simulation of single beam characteristics

All the simulation was carried out using the CyberKnife-dedicated planning System, Multiplan version 3.5.4. The characteristics of a CyberKnife beam were simulated in a virtual water phantom using the Ray and MC algorithms incorporated in the Multiplan planning system. The virtual water phantom was constructed to be 15 × 15 cm^2^ laterally and 13 cm vertically. Nine beams of different sizes, collimated by fixed collimators (sizes of 5 mm, 7.5 mm, 10 mm, 15 mm, 20 mm, 30 mm, 40 mm, 50 mm, and 60 mm) were sequentially irradiated onto the virtual water phantom at a source-to-surface distance of 75 cm. The central axis depth dose curve, OCR profile at the 5-cm depth plane, and the output factor for each irradiated beams were calculated using both the Ray and MC algorithms. The output factor for each irradiated beams was also estimated by the ratio of the central 5-cm point dose to that for the reference 60-mm beam. The Ray and MC simulated results were then compared to each other and also to each corresponding measurement results.

### Treatment plans for the head phantom

A CT scan of an anthropomorphic head phantom containing a natural human skeleton and a spherical target was used in the phantom planning study. Two distinct vGTVs were delineated along axial slices of the CT scan. The first vGTV was outlined by a contrast difference between the spherical target and the surrounding material. The second vGTV was uniformly contracted by 10 mm relative to the first target delineated. The virtual GTVs were 35 mm and 15 mm in diameter, respectively. The dose prescribed to each vGTV was set equal as 300 cGy.

Although beams of different sizes are commonly be included in a single CyberKnife treatment plan [15, 23], the same-sized beams collimated by a certain-sized fixed collimator were only used in the head phantom planning step to investigate the field sizes dependence on the Ray and MC calculated dose difference. The nominal beam sizes or fixed collimator sizes used were 5–35 mm for the larger vGTV and 5–20 mm for the smaller vGTV, respectively. Hereafter, the treatment plans for the larger and smaller vGTVs will be denoted as “*xL*” and “*xS*”, respectively, where the “*x*” represents the fixed collimator or nominal beam size in mm, e.g., a plan in which the 5-mm fixed collimator was applied to the larger vGTV will be denoted “*5L*”.

The treatment plan for the head phantom was optimized only using the Ray algorithm so that the prescribed isodose line conformally encompassed the target volume. No constraints on the number of beams and MUs per fraction were applied during the phantom planning step, although such constraints are common in actual patient planning, as discussed further below. The final dose distributions for each plan were recalculated using the Ray and MC algorithms while holding the beam configuration fixed.

### Gamma evaluation

The dosimetric accuracy of the CyberKnife treatment plan applied to the anthropomorphic head phantom was validated based on the gamma index analysis using the Gafchromic EBT-II film [14, 24]. The dose was first calibrated against the optical density of the EBT-II film across a 14-point irradiation intensity series, from 35 cGy to 490 cGy. The irradiated films were scanned in 48 bit RGB format using the EPSON 10000XL flatbed scanner. The optical density was read only from the red channel of the scanned film image.

Film measurements were then performed using the six selected plans applied to the anthropomorphic head phantom (*5 S, 10 S, 20 S, 5 L, 10 L*, and *20 L* plans). A piece of the EBT-II film was inserted into the central plane of the spherical target during the planned beam irradiation. The gamma index analysis was performed using the commercial OmniPro-I'mRT software (IBA dosimetry, Germany). The measured dose distribution for each plan was compared to each simulated dose distributions calculated using the Ray and MC algorithms, respectively, under the 2 mm/3% gamma acceptance criterion. The gamma agreement between the two simulated (Ray and MC) dose distributions for each plan was also evaluated under the same acceptance criterion, for reference.

### Actual patient planning

The 77 actual treatment plans for quasi-homogeneous brain tumor cases were only selected here to, as much as possible, exclude the tissue heterogeneity effect in the present analysis. The grossly visible tumor volume seen in the fused MR/CT scan set for each patient was contoured and defined as the gross tumor volume (GTV). The PTV was then obtained by expanding the GTV by 1 mm in all spherical directions. The doses were prescribed to PTV with different dose regimens, in the range 16–60 Gy over 1–5 fractions (Fx), with consideration for the pathology, tumor size, tumor location, and performance status.

The treatment plans were designed to meet the following requirements: (i) the entire GTV, and (ii) at least 95% of the PTV must be covered by the prescribed dose surface, respectively, (iii) the conformity index for the PTV, on the basis of the RTOG definition [25], was as close as possible to unity and did not exceed 1.20, (iv) the dose gradient beyond the PTV decreased as steeply as possible, and (v) the doses exposed to critical organs did not exceed their tolerance limits. In addition to the above requirements, the MUs/Fx and the number of beams in a treatment plan were constrained so as not to exceed 10000 MUs/fx and 200 beams, respectively, in order to avoid excessively long treatment times.

All actual treatment plans for the selected patients were originally calculated using the Ray algorithm only, but here the plans were recalculated using the MC algorithm while keeping all planning parameters the same.

### Dose evaluation

The dosimetric difference between the calculated Ray and MC distributions obtained from each treatment plan were primarily evaluated according to the mean PTV dose difference (Δ) between the Ray and MC calculations, as

$$\Delta =\frac{2(\Delta_{\text{MC}}-\Delta_{\text{RAY}})}{\left(\Delta_{\text{MC}}+\Delta_{\text{RAY}}\right)}\times 100(\%),$$

where D_*MC*_ and D_*Ray*_ represent the mean PTV doses in the MC and Ray calculations, respectively. The relationships between the dose differences (Δ) and the various planning factors, such as the average tumor diameter (*D_avg_*), effective field size (*FS_eff_*), and fill fraction (ν), were investigated. The *D_avg_* was defined as the diameter of the equivalent sphere having the same volume as the PTV. The *FS_eff_* was defined as the weighted effective field size of the beams used in the plan, while weighing the contributions of the individual beams to the target dose according to

$${\text{FS}}_{\text{eff}}=\sum [(D_{i}{\text{FS}}_{i})\cdot {\text{FS}}_{i}]/\sum \left[(D_{i}{\text{FS}}_{i})\right],$$

where *FS_i_* is the nominal size of the *i*-th beam and *D_i_* is the central axis point dose on the reference plane for the *i*-th beam calculated by multiplication of the output factor, the MUs, and the tissue phantom ratio for the *i*-th beam. The fill fraction (ν) was defined as the ratio between the *D_avg_* and *FS_eff_*, *i.e*., *ν = D_avg_/FS_eff_*. The correlations of the dose differences between the Ray and MC calculations and the above three factors were quantified using the determinant coefficient (R^2^) obtained from a linear regression method.

## CONCLUSIONS

The present study examined the use of the advanced MC algorithm in the CK system, which yielded inaccurate and overestimated doses when applied to a homogenous or a quasi-homogeneous environment compared to the simple Ray algorithm. Thus, the MC algorithm may not be recommended to tumors in homogeneous regions, such as brain tumors. It should be emphasized, however, that the present results may only be valid in the context of homogenous tissues. If assuming a heterogeneous tumor, the MC algorithm could be applied with taking into consideration of its dosimetric advantages in heterogeneous media and disadvantages in homogenous media. Even under heterogeneous circumstances, however, the use of a large number of small fields may not be recommended because this approach tends to amplify the errors in the MC calculations.
